# Causes and Consequences of Hypertriglyceridemia

**DOI:** 10.3389/fendo.2020.00252

**Published:** 2020-05-14

**Authors:** Chris J. Packard, Jan Boren, Marja-Riitta Taskinen

**Affiliations:** ^1^Institute of Cardiovascular and Medical Sciences, Glasgow University, Glasgow, United Kingdom; ^2^Department of Molecular and Clinical Medicine, Institute of Medicine, University of Gothenburg, Gothenburg, Sweden; ^3^Research Programs Unit, Clinical and Molecular Metabolism, University of Helsinki, Helsinki, Finland

**Keywords:** lipid, metabolism, VLDL, chylomicron, apoB

## Abstract

Elevations in plasma triglyceride are the result of overproduction and impaired clearance of triglyceride-rich lipoproteins—very low-density lipoproteins (VLDL) and chylomicrons. Hypertriglyceridemia is characterized by an accumulation in the circulation of large VLDL-VLDL_1_–and its lipolytic products, and throughout the VLDL-LDL delipidation cascade perturbations occur that give rise to increased concentrations of remnant lipoproteins and small, dense low-density lipoprotein (LDL). The elevated risk of atherosclerotic cardiovascular disease in hypertriglyceridemia is believed to result from the exposure of the artery wall to these aberrant lipoprotein species. Key regulators of the metabolism of triglyceride-rich lipoproteins have been identified and a number of these are targets for pharmacological intervention. However, a clear picture is yet to emerge as to how to relate triglyceride lowering to reduced risk of atherosclerosis.

## Introduction

The metabolism of triglyceride and cholesterol is intertwined because of the shared physicochemical properties of these molecules. Both need to be transported through the aqueous medium of blood plasma from sites of production or storage to tissues that require them for cell functions or energy production. Plasma lipoproteins with their hydrophobic interior and amphipathic surface provide the means of solubilizing these lipids and facilitating their passage through the circulation. The fate of the contained lipid is directed by proteins on the particle surface that interact with key lipolytic enzymes and cell membrane receptors. It is recognized that the levels of plasma triglyceride and cholesterol in most populations are far in excess of the concentrations needed to support physiological processes and this leads to pathological consequences such as atherosclerotic cardiovascular disease (ASCVD) and pancreatitis (1–3). The case for cholesterol-carrying low-density lipoprotein (LDL) as a causal factor for ASCVD is well made (4, 5) but until recently there has been significant doubt as to the role of raised plasma triglyceride, carried in chylomicrons and very-low density lipoproteins (VLDL) (collectively termed triglyceride-rich lipoproteins, TRL), as a risk factor for the development of atherosclerosis. Epidemiological studies in the general population consistently demonstrate a strong, positive association of plasma triglyceride levels with risk of ASCVD (2, 3, 6, 7) but this has been considered confounded, mainly by the link between higher levels of TRL and decreased concentrations of high-density lipoproteins (HDL), a lipoprotein class with putative cardio-protective properties (3, 8). A resurgence of interest in triglyceride as a causative agent for ASCVD and as a possible target for intervention followed reports that common allelic variations in genes regulating specifically triglyceride metabolism are associated with differences in cardiovascular disease outcomes (9–11), thus addressing the confounding issue, and that lowering plasma triglyceride is associated with reduced risk of a major cardiovascular event (12) (but may not be the whole explanation for the benefit seen in that trial).

Chylomicrons and VLDL have as their major structural apolipoprotein (apo) B48 and apoB100, respectively. Both TRL are subject to extensive remodeling during intravascular lipolysis that leads in the case of chylomicrons to the generation of “remnants” and for VLDL to a range of products including remnants within the VLDL density interval, intermediate density lipoproteins (IDL) and LDL. Given that triglyceride itself is not found deposited in atherosclerotic plaque, the influence of raised levels of this lipid on ASCVD is likely to be indirect, that is through the impact of hypertriglyceridemia on the metabolism of cholesterol-carrying lipoproteins (5, 13–15), and possibly also by activation of inflammatory mechanisms (7, 10, 16, 17). Interventions that lower triglyceride may be understood best, in terms of their impact on ASCVD risk, in light of the consequent changes in the entire apoB-containing lipoprotein spectrum. In this context, it is important to note the findings of a recent study in which genetic variants that lower plasma triglyceride were associated with reduced ASCVD risk only when there was a commensurate decrease in apo B (18). Reduced levels of high-density lipoprotein (HDL) which are a further consequence of raised triglyceride levels may contribute to the enhanced risk (3) but it is unclear whether a change in HDL attendant on triglyceride lowering will result in decreased CVD risk. Mendelian randomization studies do not support a causative link and outcome trials targeting HDL have failed to show a risk reduction (3, 8, 19).

This review explores the complex interrelationship between the metabolism of chylomicrons and VLDL, the underlying causes of hypertriglyceridemia, and the perturbations in this condition in the structure and metabolism of apoB-containing lipoprotein species. The aim is to provide a framework that not only helps in understanding the clinical sequelae of moderate to severe hypertriglyceridemia but also acts as an aid in interpreting the results of published (12) and upcoming (20, 21) triglyceride-lowering outcome trials. For LDL cholesterol (LDLc) lowering studies, systematic meta-analyses (22) reveal a straightforward relationship between the absolute reduction in LDLc and the percent decrease in ASCVD risk—a 22% relative risk reduction for each 1.0 mmol/l drop in LDLc. With the complexity inherent in TRL metabolism, it is predictable that the relationship between change in triglyceride levels and outcome will not be so simple.

## The Nature of Hypertriglyceridemia

It is recognized from studies of aboriginal, hunter-gatherer societies (1–3) and age-related lipid changes in Western populations (2, 23, 24) that the optimal plasma triglyceride concentration appears to be in the range below 1.2 mmol/l/l (<100 mg/dl) (2, 3). In developed countries the average plasma triglyceride level doubles from early adulthood (mean triglyceride 0.8 mmol/l at about 20 years) to mid-adult life (mean of 1.5 mmol/l at about 50 years old) (23, 24). This rise is driven, at least in part, by the age-related increase in body weight and adiposity since there is a strong link between being overweight or obese and higher rates of hepatic triglyceride synthesis and VLDL secretion (25, 26). Conversely, weight reduction leads to triglyceride lowering and a decrease in the production of VLDL (2, 3, 25, 26). Pregnancy is another physiological condition linked to the development of hypertriglyceridemia; VLDL levels rise several-fold over the course of gestation (27, 28), likely as a result of an action of estrogen on VLDL assembly and secretion (28) in order to deliver triglyceride to the placenta. In some women, possibly due to genetic predisposition or a metabolic disorder, frank hypertriglyceridemia can develop especially during the third trimester (27, 28).

### Accumulation of Large VLDL and Remnants in Hypertriglyceridemia

**Figure 1:**
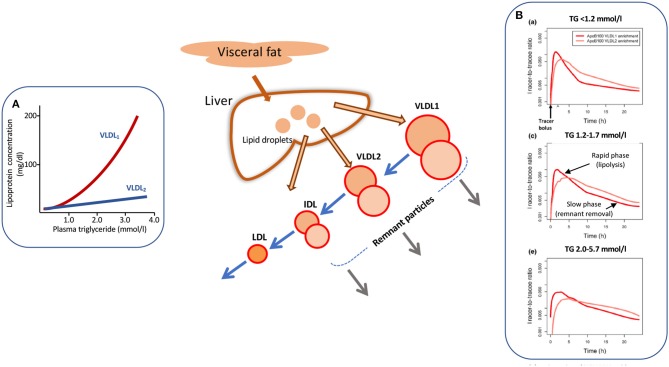
Apolipoprotein B 100 metabolic heterogeneity in hypertriglyceridemia. Elevation in plasma triglyceride is associated with an increased concentration of large VLDL-VLDL_1_ **(A)**. VLDL_1_ once secreted from the liver enters a delipidation cascade leading to the formation of smaller VLDL_2_, IDL, and LDL (main diagram). Kinetic investigations reveal metabolic heterogeneity within the delipidation pathway. As shown in **(B)** [taken from Björnson et al. (29)], a tracer of deuterated leucine administered at time 0 h appears rapidly in VLDL_1_ and VLDL_2_. Decay curves in both fractions have an initial rapid phase reflecting lipolysis and a second, slower phase due to remnant removal. This metabolic heterogeneity (as depicted by the two circles in each lipoprotein class in the main diagram) is more evident as plasma triglyceride rises **(B)**. For further detail see Packard and Shepherd (13), Björnson et al. (29), Shepherd and Packard (30), and Packard et al. (31).

Increased levels of plasma triglyceride are associated with the accumulation of large, triglyceride-rich VLDL-VLDL_1_–particles with a diameter range of 50–80 nm containing about 70% triglyceride by mass. Smaller VLDL-VLDL_2_–which are 30–50 nm in diameter and consist of about 30% triglyceride show a moderate elevation (Figure 1A) (13, 30, 32, 33). The liver has the capacity to vary the amount of lipid loaded onto the growing lipoprotein particle in the endoplasmic reticulum, and indeed depending on triglyceride availability can assemble and secrete particles that range in size from VLDL_1_ to LDL (13, 30, 32–35). A number of factors determine the assembly and secretion rate of apoB100 lipoproteins including hormone levels, intracellular lipid trafficking, protein regulatory factors (34, 35), and possibly proprotein convertase/subtilisin kexin type 9 (PCSK9) (36). VLDL_1_ synthesis is driven by the supply of triglyceride from intracellular stores, *de novo* lipogenesis, fatty acid uptake, and hepatic chylomicron remnant removal (37, 38). Overproduction of smaller VLDL_2_, on the other hand, is linked to raised cholesterol levels (39) and is a feature of familial hypercholesterolemia (40, 41). Kinetic investigations have demonstrated that the metabolic fate of circulating VLDL particles is a function of their size and lipid and apoprotein composition (13, 30, 33, 42), especially their apoE and apoCIII content (42). So, an understanding of the causes and consequences of hypertriglyceridemia needs to encompass the factors that govern lipoprotein assembly in the liver and the enzymes and receptors that regulate flow down the VLDL-LDL delipidation cascade (Figure 1, main diagram).

As plasma triglyceride rises in the population, metabolic abnormalities appear throughout the VLDL_1_-VLDL_2_-IDL-LDL delipidation cascade (Figure 1). At triglyceride levels <1.2 mmol/l there are approximately equal amounts of VLDL_1_ and VLDL_2_ in the circulation (inset A) and kinetic studies show that there is both a low secretion rate and rapid clearance of VLDL_1_ (13, 30, 32). As plasma VLDL_1_ rises due to a combination of overproduction and less efficient lipolysis, there is increased metabolic heterogeneity with the appearance of slowly metabolized species in VLDL_1_ and VLDL_2_ (13, 14, 25, 30, 32, 38–43). These products of inefficient VLDL delipidation are considered “metabolic remnant particles” (Figure 1). They accumulate in proportion to the increase in plasma triglyceride (7, 10, 15) and are believed to be able to contribute to the deposition of cholesterol in atherosclerotic lesions (15, 44) as well as promote inflammatory processes (16). By way of illustration, Figure 1B shows the heterogeneity seen in apoB100 metabolism in VLDL_1_ and VLDL_2_ in subjects with low, average, and elevated plasma triglyceride concentrations [taken from Björnson et al. (29)]. It can be seen that that once peak enrichment is achieved at about 2–5 h after injection of the tracer (deuterated leucine), there is a biphasic log-linear decay indicative of the presence of at least two metabolically distinct lipoprotein species. The initial rapid phase is linked to lipolysis while the later, slower decay is likely attributable to remnant particle clearance. In the group of subjects with optimal plasma triglyceride, the slow component was a minor contributor to overall catabolism but in the groups with higher triglyceride, its contribution in both VLDL_1_ and VLDL_2_ became more pronounced. Similar kinetic features are observed when apoB metabolism is followed in the total VLDL fraction in hypertriglyceridemic and hypercholesterolemic subjects (13, 14, 25, 41, 45–47).

### Impact of Hypertriglyceridemia on VLDL-LDL Metabolic Pathways

**Figure 2:**
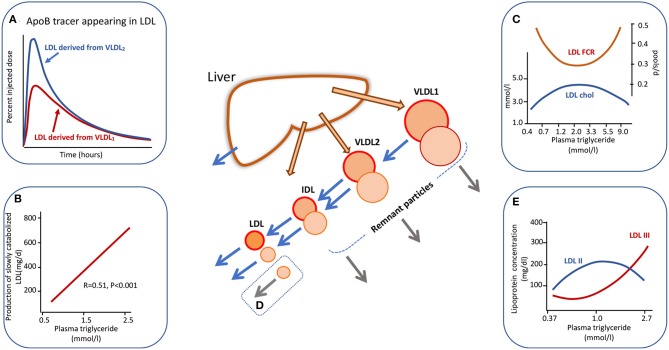
Apolipoprotein B100 metabolic cascade in hypertriglyceridemia. Plasma triglyceride levels influence the metabolism of LDL as an extension of the metabolic heterogeneity seen in Figure 1. LDL is derived from the delipidation of VLDL_1_ but the extent of conversion is less than for VLDL_2_. The LDL (followed by virtue of the radioactive label attached to VLDL_1_ apoB) from VLDL_1_ has a slow catabolic rate **(A)**. LDL derived from VLDL_2_ delipidation exhibits a two-phase clearance curve. The first part represents LDL with a rapid clearance rate (presumably derived from directly secreted VLDL_2_) while the second phase exhibits the same clearance rate as LDL derived from VLDL_1_. LDL kinetic studies show a positive relationship between the amount of slowly catabolised LDL produced and the plasma triglyceride concentration across the “normal range” **(B)**. Plasma triglyceride across the full range of normal through to severe hypertriglyceridemia exhibits a complex relationship to LDL cholesterol concentration and to LDL fractional catabolic rate (FCR) as shown in **(C)**. Between triglyceride levels of 0.5 to about 3.0 mmol/l FCR falls and LDL concentration rises due to increased production of VLDL_1_ which is converted to slowly metabolized LDL. In severe hypertriglyceridemia) (>5.0 mmol/l), LDL FCR is increased and the concentration decreases due to rapid clearance by stimulated receptor-independent routes **(D)**. **(E)** Shows the change in LDL size profile as plasma triglyceride increases. For further detail see Ference et al. (4), Borén et al. (5), Packard and Shepherd (13), Packard et al. (31), and Caslake et al. (48).

Heterogeneity in apoB metabolism in hypertriglyceridemia is not confined to the VLDL density range. In a series of experiments using radiolabelled lipoproteins as tracers, we found that when VLDL_1_ and VLDL_2_ were isolated, labeled with alternate iodine isotopes (^131^I, ^125^I), and injected into the donors the metabolism of these two subfractions differed. There appeared to be “metabolic channeling” within the VLDL-LDL delipidation pathway (13, 30, 39); that is VLDL_1_ and VLDL_2_ had distinct rates of conversion to IDL and LDL, and there was variation in the extent of direct catabolism of remnants from the VLDL_1/2_ and IDL density intervals as depicted in the central diagram of Figure 2. This observation is significant since it implies that the pedigree of an LDL particle influences its metabolic properties and hence its potential atherogenicity. Figure 2A shows the appearance and disappearance curves for LDL derived from VLDL_1_ and VLDL_2_ (13). The latter was rapidly and more completely converted to LDL while lipolysis of the former subfraction generated less LDL. LDL formed from ^125^I-VLDL_2_ (which of course will include directly secreted VLDL_2_ and the products of VLDL_1_ lipolysis) exhibited two catabolic phases; an initially rapid rate followed by much slower decay whereas LDL formed from ^131^I-VLDL_1_ delipidation was catabolized only at the slower clearance rate (Figure 2A).

The presence of metabolic heterogeneity in the LDL density range linked to plasma triglyceride levels was examined further using radiolabelled LDL tracers (31, 48, 49). It was found that as plasma triglyceride rose across the normal range the LDL fractional catabolic rate (FCR) fell and LDL concentration increased until a triglyceride value of about 2–3 mmol/l (Figure 2C). Above this level the LDL FCR then paradoxically rose as triglyceride increased, and LDL concentration fell. This positive association of LDL FCR with the severity of hypertriglyceridemia has been reported consistently in the literature (14, 50–53). An explanation that has been offered for the metabolic heterogeneity depicted in Figures 2A–C is that the conformation of apoB100 on LDL particles differs depending on their pedigree (13, 31); in LDL originating from VLDL_2_/IDL secreted directly from the liver apoB100 has a conformation that is able to bind well to LDL receptors and hence the particle can be catabolized efficiently, whereas LDL derived from VLDL_1_ delipidation contains apoB100 in which the receptor binding site is less well-expressed and its clearance is impeded. In analyzing a series of LDL kinetic studies, we observed a positive relationship between plasma triglyceride level across the normal range and the amount of slowly metabolized LDL produced (Figure 2B) (31, 48, 49). This phenomenon potentially explains the fall in overall LDL FCR as plasma triglyceride rises across the normal range from 0.4 to 2.0 mmol/l (Inset C). The accelerated catabolism of LDL in severe hypertriglyceridemics (i.e., at plasma triglyceride levels above 5 mmol/l in Figure 2C) has been attributed not to enhanced LDL receptor activity but to stimulation of receptor-independent pathways (Inset D) (13, 14, 51), possibly involving the reticuloendothelial system.

### LDL Structural Abnormalities in Hypertriglyceridemia

LDL subfraction distribution is also affected markedly by plasma triglyceride levels (Figure 2E). It has been shown by a number of investigators that the concentration of small, dense LDL (LDL-III) increases significantly when triglyceride rises above about 1.5 mmol/l and there is a reciprocal drop in LDL-II, the most abundant subfraction in normal subjects (5, 13, 31, 52, 53). Models for the formation of small, dense LDL link its metabolic and structural heterogeneity (13, 31, 52, 53). In the scheme depicted in Figure 2 [and explained in more detail in (13, 31)], it is postulated that slowly metabolized LDL derived from the lipolysis of large VLDL species (VLDL_1_) has a prolonged residence time in the circulation and this allows extensive inter-particle transfer to take place via the agency of cholesteryl ester transfer protein (CETP). Cholesteryl ester is removed from the core of LDL particles and replaced with triglyceride from VLDL donor particles. This compositional change makes LDL susceptible to hepatic lipase action. This enzyme hydrolyses surface phospholipid and core triglyceride to generate a smaller particle. Higher levels of plasma triglyceride, therefore, not only favor formation of slowly cleared LDL (Figure 2) but also provide an abundant supply of triglyceride to drive CETP-mediated exchange. Since small, dense LDL has been proposed to be more atherogenic than its normal-sized counterpart due to enhanced binding to arterial wall proteoglycans and its ability to be oxidized (44, 52–54), this conceptual model provides one of the key links between variation in plasma triglyceride and ASCVD risk.

From the above it is clear that elevation in plasma triglyceride is accompanied by metabolic and structural perturbations throughout the apoB100-containing lipoprotein spectrum. Since disadvantageous changes start to be evident as low as 1.5 mmol/l, this has been used as a cut-off for “borderline” hypertriglyceridemia while “moderate” and “severe” forms of the condition are defined by plasma triglyceride concentrations in the range 2.3–5.0 and >5.0 mmol/l, respectively (2, 3). What is obvious from a consideration of these metabolic patterns is that hypertriglyceridemia is not a uniform disorder (in contrast to hypercholesterolemia which is due primarily to a relatively simple elevation in LDL), rather it is accompanied by a spectrum of metabolic alterations and the phenotypic presentation will depend on the activities of a range of key regulatory factors.

## Genetic, Hormonal and Dietary Regulation of Plasma Triglyceride

Some of the most illuminating investigations into the factors that control VLDL and LDL metabolism have come from the study of inherited conditions where a specific enzyme, receptor or cofactor is absent or deficient.

### Role of Lipases in Metabolism of Triglyceride-Rich Lipoproteins

**Figure 3:**
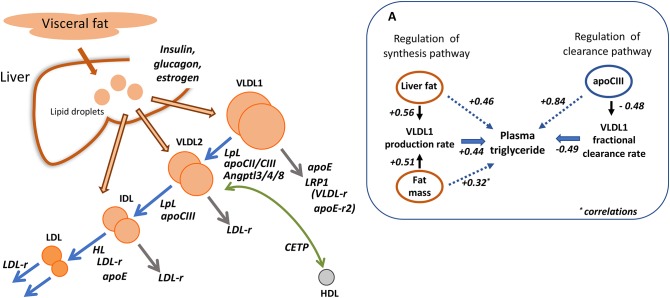
Regulation of apolipoprotein B metabolism by key enzymes, receptors and cofactors. The main diagram depicts the known roles of lipoprotein and hepatic lipase (LpL, HL); cholesteryl ester transfer protein (CETP); apolipoproteins CII, CIII, and E; LDL receptor (LDL-r). The potential role of the VLDL receptor (VLDL-r), apoE receptor 2 (apoE-r2), and LDL receptor related protein 1 (LRP1) in VLDL_1_ removal is speculative. **(A)** Shows the relative quantitative importance of liver and body fat in regulating VLDL_1_ production and of apoCIII in controlling VLDL_1_ clearance [adapted from (43)]. The numerical values quoted are correlation coefficients.

Familial chylomicronemia syndrome, characterized by the accumulation of extremely high levels of chylomicrons and in many cases VLDL is the result of inherited deficiencies in key factors in the pathways of TRL assembly and clearance (55). One of the best characterized of these disorders is absence of functional lipoprotein lipase (LpL) activity (2, 3, 55–57) in which VLDL catabolism is severely impaired, in particular the lipolysis of VLDL_1_ (Figure 3) (56, 57). In experiments using tracers of radio-iodinated VLDL_1_ and VLDL_2_ in subjects with the condition, it was observed that the FCR of VLDL_1_ was decreased 10-fold but apoB production into this lipoprotein fraction was similar to normal. VLDL_2_ clearance, on the other hand, was not significantly impaired by lack of LpL (57). Deficiency of the other key lipase in lipoprotein metabolism—hepatic lipase (HL)—is associated with hypertriglyceridemia but not to the same extent as seen in LpL deficiency (58–60). Investigations reveal that this enzyme is more active at hydrolyzing lipid in smaller particles in the VLDL_2_, IDL, and LDL density intervals. HL deficient subjects exhibit reduced clearance rates for VLDL and IDL, and slowed conversion of VLDL_2_ to IDL and IDL to LDL (58–60) (Figure 3). In the subject we studied there was an almost complete inability to convert IDL to LDL (58).

### Lipoprotein Receptors in VLDL Metabolism

The role of the LDL receptor in apoB metabolism was thought initially to be limited to facilitation of LDL clearance. However, kinetic investigations in homozygous familial hypercholesterolemia (FH) indicate that VLDL and IDL metabolism are affected also (40, 41, 47). While the production and clearance of the largest VLDL particles—VLDL_1_ (Figure 3)—are not altered in homozygous FH, there is overproduction of VLDL_2_ [and total VLDL where this was studied (41)], and delayed clearance of this lipoprotein class, a reduced IDL FCR, and a very slow conversion of IDL to LDL (taking an average of 100 h in affected individuals compared to about 20 h in normal subjects) (40, 47). Understanding the full contribution of the LDL receptor to apoB metabolism is important when elucidating the mechanism of action of drugs that stimulate its activity such as statins and PCSK9 inhibitors (see below). This was explored further in experiments in which unmodified VLDL subfractions and their chemically altered counterparts (with blocked arginine resides) which could not bind to receptors were labeled with the two forms of radioiodine and administered to normal volunteers (61). It was observed that modification did not impact on the kinetics of large VLDL (VLDL_1_) but delayed the processing and clearance of a smaller VLDL (VLDL_2_)/IDL fraction as well as the catabolism of LDL. These findings suggest again that the LDL receptor is not involved in VLDL_1_ metabolism, at least in normal subjects, but plays a part in the catabolism of VLDL_2_ and IDL particles as well as LDL (Figure 3). Since the modified VLDL_2_/IDL fraction exhibited a slower conversion rate to LDL, this was taken as further evidence that the LDL receptor contributed to this process. The agreement between these studies and the observations of the metabolic disturbance in FH where there is an inherited deficiency of LDL receptors is striking (Figure 3). Direct removal of VLDL_1_ may be facilitated by other members of the LDL receptor superfamily which include LDL receptor related protein 1 (LRP1), the VLDL receptor, and the apoE-receptor 2 (62, 63). LRP1 is a prime candidate since it binds apoE and has been implicated in the hepatic uptake of chylomicron remnants which share many of the structural features of partially lipolyzed VLDL_1_ particles (63) (Figure 3).

### Role of Apolipoproteins CIII and E in Metabolism of Triglyceride-Rich Lipoproteins

Apolipoproteins CII, CIII, and E are small proteins carried on the surface of lipoproteins that influence their metabolic properties. These apoproteins are not distributed uniformly and are found in different sub-types of particles within each density range (42). The metabolism of apoB100-containing lipoproteins with and without apoCIII and apoE has been investigated in detail and related to risk of ASCVD (42, 64). Other studies have delineated the metabolic consequences of inheriting the various apoE isoforms—apoE2, E3, and E4 (65, 66)—or a deficiency in apoCIII (67–69). In our study of subjects homozygous for the three apoE protein variants (65) it was found that those who were E2/2 had a reduced rate of VLDL_1_ and VLDL_2_ direct catabolism indicating delayed remnant removal, and a marked reduction in the rate of conversion of IDL to LDL (Figure 3). ApoE isoform type was a major influence on the extent of conversion of VLDL_2_ to LDL; this was 25% in E2/2, 50% in E3/3, and 70% in E4/4 subjects (65). Even a single copy of the apoE4 is sufficient to influence apoB kinetics leading to an increased VLDL to LDL conversion and a reduced LDL FCR (66). These observations help explain why apoE is the most important inherited trait influencing plasma cholesterol levels, and why in E2/2 individuals with hypertriglyceridemia, remnant lipoproteins accumulate throughout the VLDL-IDL density range (1–3). This “remnant” or Type III hyperlipidemia is associated with accelerated atherosclerosis even though LDL levels are generally low.

It has long been suspected that apoCIII is a major regulator of plasma lipid concentrations. Its role in triglyceride transport and as a potential intervention target has been reviewed recently (67). Overproduction of apoCIII is associated with raised triglyceride levels (70), and this apoprotein is a major determinant of VLDL apoB100 and VLDL triglyceride clearance rates (Figure 3) (67–72). There appear to be a number of ways in which apoCIII can influence triglyceride metabolism. It has reported to enhance VLDL assembly and secretion in the liver, inhibit the action of lipoprotein lipase and hence slow VLDL lipolysis, and possibly also retard the direct clearance of VLDL remnants by receptors (67–72). VLDL particles separated into various sub-types on the basis of apoprotein content have been shown to be removed significantly less efficiently from the circulation if they carry apoCIII (42). Interest in apoCIII as a potential intervention target was reinforced by the finding that inherited loss-of-function mutations in this protein were associated with low plasma triglyceride and a reduced risk of CVD (9–11, 67). Further exploration of the metabolic consequences of being heterozygous for apoCIII deficiency (69) gave rise to the observation that compared to their unaffected siblings, affected subjects had about half the apoCIII in plasma. VLDL apoB production was the same both groups (suggesting no impact on VLDL assembly) but the clearance rate was doubled in carriers of the apoCIII mutation mainly due to a more efficient VLDL to LDL conversion rather than stimulation of VLDL direct clearance. Other proteins including apoA5 (73), Angptl3, Angptl4, and Angptl8 (74–76) have been identified as important in regulating lipolysis, and linked to variation in plasma triglyceride and CVD risk (11).

### Impact of Obesity and Hormones on Triglyceride Metabolism

Secretion of apoB-containing particles from the liver is governed by the body's requirements for triglyceride and cholesterol in peripheral tissues and the need to regulate the amount of lipid stored in hepatocytes since pathological consequences can ensue (such as non-alcoholic fatty liver disease and hepatosteatosis) when intracellular fat accumulates (77). Increased VLDL synthesis is present in non-obese and obese subjects who have high levels of liver fat (25, 26, 32, 38, 78, 79), and we observed positive correlations between the amount of body fat mass and liver fat with both VLDL_1_ apoB and VLDL_1_ triglyceride production rates (Figure 3A) (43). Likewise, in obese subjects on a weight-loss diet, the decrease in VLDL secretion rate is related to the fall in liver fat (25, 26, 79).

Plasma triglyceride concentration across the normal range and in the hypertriglyceridemic population is (as set out above) primarily a function of the level of VLDL_1_ which in turn is governed by the relative rates of production and clearance (lipolysis and direct catabolism) of this particle. In a recent multicenter investigation in which it was possible to include a large number of subjects with abdominal obesity, the quantitative contributions of key regulatory factors were assessed in a statistical model of the determinants of plasma triglyceride (43). VLDL_1_ -apoB and -triglyceride kinetic parameters explained about 70% of the variation in plasma triglyceride and, as depicted in Figure 3A, significant correlations were found between liver fat/ body fat mass and the VLDL_1_ synthesis rate, while the VLDL_1_ fractional clearance rate was inversely related to plasma apoCIII concentration. Lipoprotein and hepatic lipase activities, and apoCII and apoA5 levels were included in the model but none of these exhibited a significant association with VLDL_1_ metabolic parameters. As in earlier analyses, overall it is the clearance rate that is the dominant feature in regulating VLDL accounting for about 70% of the variation while production rates explain around 25% (13, 43).

Hormones associated with glucose and lipid homeostasis are also important regulatory factors. The presence of insulin resistance is positively associated with VLDL_1_ synthesis rates in both normoglycemic and type 2 diabetic subjects (32, 78–80) while glucagon acts to suppress VLDL triglyceride release (81). In contrast, there seems to be no relationship between indices of insulin resistance or the presence of type 2 diabetes and direct VLDL_2_ production (78, 81). In patients with type 2 diabetes, it can be seen that VLDL- triglyceride and-apoB production rates were positively related to the extent of hyperglycaemia (even in well-controlled patients) (78, 79). Further, there was no difference in particle composition between diabetic and non-diabetic subjects indicating that the hypertriglyceridemia seen in type 2 diabetes is associated with the overproduction of VLDL_1_ particles of normal size and composition. The direct influence of insulin on VLDL metabolism can be seen also in experiments in which the hormone was administered acutely to normal and diabetic subjects (79, 80); the former showed suppression of VLDL_1_ (but not VLDL_2_) production while the latter showed no such effect (80). These results support the view that VLDL_1_ and VLDL_2_ assembly and secretion are independently regulated in the liver and that the metabolism of VLDL_1_ due to its role in triglyceride transport is integrated into whole body energy homeostatic control.

### Impact of Dietary Constituents on Triglyceride Metabolism

Both quantity and composition of the diet impact on plasma triglyceride concentrations and can lead to borderline or moderate hypertriglyceridemia. Excessive intake of calories leads to storage of triglyceride in adipose and other tissues. Of particular concern for the regulation of VLDL metabolism is the accumulation of visceral and liver fat. VLDL synthesis, as noted above, is driven by the supply of triglyceride to the lipoprotein assembly pathway in hepatocytes (25, 26, 43, 78, 81, 82). Liver triglyceride is derived from a number of sources, uptake of fatty acids from the bloodstream (especially that released from visceral fat depots), release of fatty acids from stored triglyceride in cytoplasmic lipid droplets, uptake and hydrolysis of chylomicron remnants, and *de novo* lipogenesis (34, 35, 37, 78, 81, 82). These sources can be used variably by the liver during the course of fasting and feeding cycles. Of the major dietary constituents, carbohydrate intake seems to have the greatest impact on plasma triglyceride levels. Ingestion of a high carbohydrate diet (where carbohydrate accounts for >55% of energy intake and fat has been reduced to maintain caloric balance) leads to the development of raised VLDL due to an increase in VLDL-triglyceride and -apoB production rates and a decrease in clearance (83). In contrast, a carbohydrate-restricted diet leads to rapid, beneficial changes in hepatic lipid metabolism with decreased *de novo* lipogenesis, increased fatty acid oxidation, and substantial reductions in VLDL triglyceride and plasma apoCIII levels (84). Variation in the type of fat, i.e., replacing saturated fat with monounsaturated or polyunsaturated fatty acids in the diet also alters plasma triglyceride and VLDL metabolism but not to the same extent as replacement of fat with carbohydrate (85). A recent development in human nutrition has been the incorporation of increasing amounts of fructose in drinks and foodstuffs. There is now clear evidence that this sugar has significant lipogenic properties stimulating hepatic *de novo* lipogenesis and liver fat accumulation, and raising triglyceride in the circulation. Through these mechanisms, excessive consumption of fructose is linked to development of cardiometabolic disorders including metabolic syndrome of which hypertriglyceridemia is a feature (86).

## Integrated View of the Role of Liver and Intestine in Triglyceride Transport

For pragmatic as well as theoretical reasons, a great deal of the research into triglyceride metabolism has been conducted in the fasted state; “pragmatic” because the VLDL-LDL pathway arising in the liver is in steady state and can be investigated using classical kinetic techniques, “theoretical” because the vast majority of epidemiological surveys linking plasma triglyceride levels to risk of cardiovascular disease have drawn blood from subjects fasted overnight i.e., it was VLDL triglyceride that was measured. However, many researchers over the last three decades have pointed out that we spend most of our lives in the post-prandial state and the contribution of intestine-derived lipoproteins—chylomicrons and their remnants—to atherosclerosis must not be ignored (1–3, 10, 15, 87–90). Reviewing the evidence regarding the association of non-fasting plasma triglyceride concentrations to CVD risk, it was concluded by a European expert panel that this parameter was an important risk factor and possibly superior to assay of fasting levels of the lipid (91).

### Investigating Chylomicron ApoB48 and VLDL ApoB100 Kinetics

Investigating chylomicron metabolism is a challenge due to its dynamic nature—these triglyceride-rich lipoproteins appear as a wave following fat meals—and the need to distinguish it from very low-density lipoprotein (VLDL) mediated triglyceride transport. A variety of approaches have been used with varying degrees of success. Retinyl palmitate has been employed in the past as a marker of intestinally derived particles (92) but most kinetic studies now use tracer techniques to assess apoB48 kinetics (since this form of apoB is found solely in particles of intestinal origin: apoB100 in VLDL is exclusively liver-derived) (92–98). However, the low abundance of apoB48 and its transient behavior have presented challenges in the conduct and interpretation of kinetic studies. Some investigators have addressed these issues by employing quasi-steady-state designs with participants fed micro-meals across the day (93, 94, 97) others by the use of simplified kinetic analysis (95) but neither of these approaches replicates the physiological situation of the overlay of a complex, non-steady state process—post-prandial lipid absorption—on the relatively constant VLDL pathway. Using the continuous feeding protocol, it was found that the production rate of apoB48 in normal subjects was about 70 mg/d which is relatively minor compared to the 500–1,200 mg/d for apoB100 in VLDL_1_ (93, 94, 97). To put this in the context of triglyceride mass transport, it can be estimated that the intestine presented with these small meals processes and releases ~90 g of triglyceride per day in the form of chylomicrons (given an average dietary intake of fat) whereas the liver secretes in the region of 25 g/d of VLDL_1_-triglyceride (13, 25, 33, 43). Since apoB48 has a molecular weight that is half that of apoB100, the production rate in mg/d can be doubled to get an idea of the relative number of particles released from both organs. Further, chylomicrons with diameters in the 100–200 nm range are able to accommodate much more triglyceride per particle than VLDL_1_ and this provides the necessary capacity for the intestine to handle the substantial amount of lipid taken up during absorption of a fat meal. The overall fractional clearance rate for apoB48 was of the order of 4 pools/d (93–95, 97) which is lower than that seen for VLDL_1_ at 5–15 pools/d indicating that while chylomicrons are the preferred substrate for lipoprotein lipase and hence hydrolysis of their core triglyceride is rapid, removal of the resultant remnant apoB48 particle is relatively slow compared to VLDL_1_ clearance.

### Integrated Regulation of Chylomicron and VLDL Metabolism

Adopting a more physiological feeding pattern in which a fat-rich meal is consumed and the resultant peak of alimentary lipaemia quantified either by measuring retinyl palmitate as tracer or quantifying apoB48 plasma levels, it was observed that triglyceride levels rose over 2–6 h in normal subjects and then returned to near fasting concentration by about 10 h (92, 96). In subjects with hypertriglyceridemia, the peak of chylomicronemia is higher and it takes much longer for the wave of particles to be cleared from the circulation and apoB48 to return to baseline (2, 3, 7, 92, 96). It has been observed that apoB48 is present in plasma after an overnight fast (before any food is ingested) in an amount that is proportional to the plasma triglyceride level (92, 96, 98). These findings indicate either that the intestine releases lipoproteins containing apoB48 on a continuous basis or that apoB48 remnant particles persist for much longer in the circulation than previously thought (29, 94, 95, 98). The latter possibility was investigated in a recent study in which plasma apoB concentration was monitored over a prolonged fast and it was seen in hypertriglyceridemic subjects that it was many hours before apoB48 reached a nadir (29, 98). Another feature of apoB metabolism is the finding that when apoB48 is measured across the whole size range of triglyceride-rich lipoproteins following a dietary fat load, it appears in the VLDL_1_ and VLDL_2_ density intervals in about the same timeframe as in chylomicrons (92, 96–98) either as a result of rapid lipolysis or by direct secretion from the intestine, that is enterocytes not only release VLDL sized particles in the fasted state but also at an augmented rate during fat absorption.

**Figure 4:**
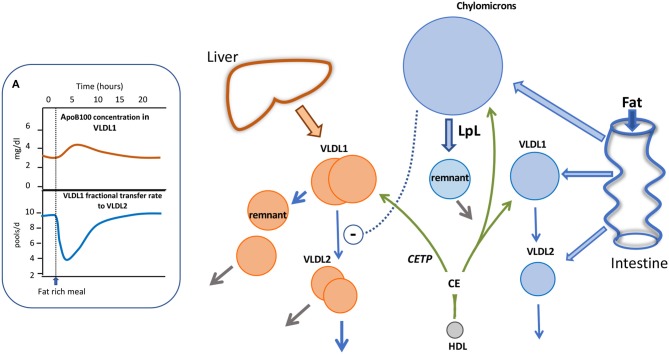
Impact of alimentary lipemia on apolipoprotein B metabolism hypertriglyceridemia. The intestine can secrete chylomicrons, and particles in the VLDL density range during lipid absorption. The appearance of chylomicrons in the circulation impairs VLDL_1_ lipolysis as shown in **(A)**. In an integrated multi-compartmental model, following a fat meal, VLDL_1_ apoB100 concentration increased and this was attributed to a drop in VLDL_1_ to VLDL_2_ transfer (i.e., reduced lipolysis rate) (29, 98). High density lipoprotein (HDL) via the agency of cholesteryl ester transfer protein (CETP) can transfer cholesteryl ester (CE) to triglyceride-rich lipoproteins thereby increasing their cholesterol content.

With the advent of more sensitive mass spectrometry techniques it has become possible to explore apoB48 kinetic behavior in much greater detail than before. By following the metabolism of trace-labeled apoB48 and apoB100 in chylomicrons and VLDL following a fat-rich meal, we have been able recently to build an integrated picture of the metabolism of liver and intestinally-derived triglyceride-rich lipoproteins during fat absorption, as depicted in Figure 4. Key findings were that in subjects with borderline to moderate hypertriglyceridemia, apoB48 metabolism was significantly perturbed. There was a low level of basal apoB48 direct secretion (prior to the fat meal) into the VLDL_1_ and VLDL_2_ density ranges and this increased several-fold post-prandially (Figure 4). This indicates that, indeed, the intestine assembles and secretes VLDL throughout the day—fasted or fed—and this pathway contributes significantly to circulating TRL found in the VLDL_1_ and VLDL_2_ density ranges. It was estimated that in subjects with raised triglyceride (>2 mmol/l) apoB48 secretion into VLDL was ~600 nmol/d compared to 2,200 nmol/l for apoB100, and across the day apoB48-containing lipoproteins accounted for about 25% of VLDL particles (29). It is known that chylomicrons and VLDL compete for the same lipolytic mechanism with the former being preferred. When chylomicrons appear, apoB100 levels in VLDL have been shown to rise sharply (29, 92, 97, 98) (Figure 4A). We were able to account satisfactorily for the rise in VLDL_1_-apoB100 by including an inhibitory action of chylomicrons on VLDL_1_ clearance; the VLDL_1_-apoB100 FCR fell transiently by an average of 60% (Inset A) and then returned to pre-meal levels once chylomicrons were cleared. Thus, it appears that not only does the intestine contribute more than previously thought to circulating TRL found in the VLDL density range, but also that the release of chylomicrons impacts on the metabolism of liver-derived VLDL, slowing its clearance from the bloodstream (as depicted by the dashed arrow in Figure 4) and thereby favoring remnant generation. Clearance rates of both apoB48 and apoB100 particles in VLDL_1_ and VLDL_2_ were retarded markedly in hypertriglyceridemic subjects with residence times rising from 1 to 4 h in individuals with low triglyceride (<1.2 mmol/l) to 4–13 h in those with triglyceride >2 mmol/l (29). It follows, therefore, that both liver and intestine are responsible for the generation of long-lived remnant particles that could promote atherogenesis in hypertriglyceridemic subjects.

## Mechanistic Insights From Pharmacological Interventions

**Figure 5:**
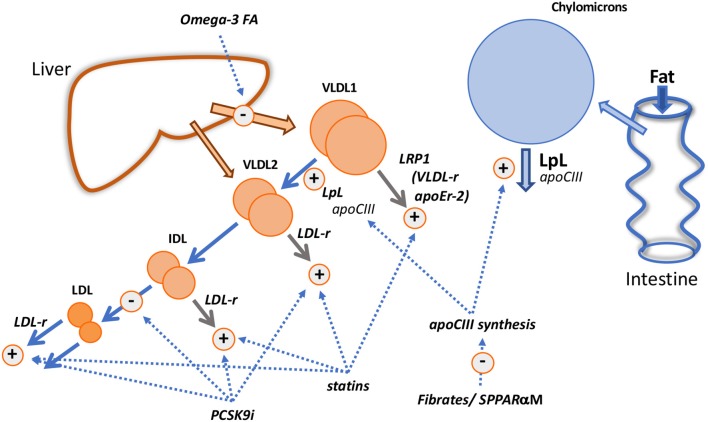
Mechanistic insights from pharmacological interventions. The diagram shows the known and putative actions of fibrates/selective peroxisome proliferator receptor α modulators (SPPARαM); fish oils (Omega-3 FA); statins; PCSK9 inhibitors (PCSK9i).

Further insight into the factors that regulate triglyceride transport comes from clinical trials in which lipid-lowering drugs have been shown to alter the metabolism of apoB100 and B48 in subjects with elevated plasma triglyceride levels (Figure 5).

### Insights From the Mechanism of Action of Fibrates and Fish Oil

Fibrates reduce plasma triglyceride levels—both in VLDL and chylomicrons—principally by increasing the FCR of VLDL (50, 99–105), especially that of the VLDL_1_ subfraction (102, 105) and reducing the extent of alimentary lipemia (100). This is likely due to the action of the drug on the PPARα response element within the apoCIII gene promoter region that results in decreased expression of the protein (1–3, 50, 100, 101) and, as a consequence, enhanced activity of lipoprotein lipase. Acceleration of VLDL transfer down the delipidation cascade may, in line with the findings in apoCIII deficiency states (67–69), be the main effect of fibrates on apoB metabolism but in those with elevated triglyceride levels direct removal of VLDL particles may become quantitatively important (Figure 1) and fibrate-induced changes in apoCIII levels on particles may alter this pathway also (42). However, we observed that bezafibrate therapy in hypertriglyceridemics increased the amount of smaller VLDL (VLDL_2_) particles in the circulation (102, 105) due to the fact that clearance of these species was not stimulated by the drug.

In individuals with initially low plasma triglyceride, fenofibrate therapy caused LDL cholesterol and apoB concentrations to fall by 10–20% (49, 50, 105) due to an increased clearance rate. Further exploration of the changes in LDL metabolism led to the finding that on fenofibrate the production rate of slowly metabolized LDL (Figure 2) was reduced and that of rapidly metabolized LDL enhanced (49, 105). Thus, the observation that in subjects with borderline hypertriglyceridemia and raised LDL cholesterol, fenofibrate therapy increased overall LDL clearance was understood better in terms of this shift in the pedigree of LDL particles, possibly attributable to the drug-induced alterations in the metabolism of VLDL_1_, the source of slowly cleared LDL (Figure 2). In severe hypertriglyceridemia, fibrates correct partially the hypercatabolism of LDL (Figures 2C,D) and LDL concentration increases by 20–30% (49–51). The decrease in clearance rate has been attributed to less active receptor-independent catabolic pathways (51, 105). Structural changes in LDL accompany these metabolic perturbations with fibrate therapy being associated with an increase in larger sized LDL subfractions (99–101, 104–106).

Omega-3 fatty acids [eicosapentaenoic (EPA) and docosahexanoic (DHA) acids and their derivatives] given in high doses (>2 g/d) lower VLDL in proportion to the dose given and the basal plasma triglyceride level; reductions vary from 20% in moderate hypertriglyceridemia to about 35% in those with more severely elevated levels (1–3, 85, 107, 108). Kinetic investigations show consistently that high dose fish oils reduce the production of VLDL triglyceride and apoB (i.e., decrease particle secretion rates) (Figure 5) without altering the overall fractional clearance rate of this lipoprotein, a mechanism of action that is clearly distinct from that of fibrates (108–112). The decrease in synthesis and secretion of VLDL on omega-3 fatty acid supplementation has been linked in animal models with decreased hepatic lipogenesis, stimulation of mitochondrial fatty acid oxidation in the liver (108, 109, 112) and enhanced degradation of apoB in the endoplasmic reticulum (112).

LDL levels have been reported to be variably affected by fish oil supplementation. LDL cholesterol exhibits usually no change or a modest increase in individuals with moderate hypertriglyceridemia but in severe hypertriglyceridemia, a substantial rise in LDL cholesterol in the order of 30% can occur (similar to the response to fibrates) (108). Investigators have found repeatedly that the extent of VLDL to LDL conversion is increased on fish oil (85, 108, 111, 113). The reason for this is unknown but is possibly not due to a change in overall lipolytic capacity since there is no change in VLDL fractional catabolic rate. Fish oils have been reported to have variable effects on lipase activity, and at least in hypertriglyceridemic subjects there appears to be no increase in lipoprotein lipase measured as heparin-releasable enzyme (112). A reduction in plasma levels of apoCIII has been seen in severe hypertriglyceridemic subjects given omega-3 fatty acids (112) and this may influence the lipolytic potential of both VLDL_1_ and chylomicrons (Figures 3, 4) but again, in contrast to the fibrate mechanism of action, an increase in VLDL FCR would have been expected if this was a rate-limiting factor. Another explanation along the lines of the concept depicted in Figure 2, is that fish oils by decreasing triglyceride availability for VLDL assembly favor the release of smaller VLDL which is more efficiently converted to the lipolytic products IDL and LDL. There have been few experiments looking at the metabolism of VLDL subfractions on fish oil. However, observations supporting this hypothesis come from a small study in patients with diabetes that found that omega-3 fatty acid supplementation (1.8 g/d EPA) led to a specific decrease in VLDL_1_ triglyceride concentration due to decreased synthesis. There was no change in VLDL_2_ triglyceride level or the kinetics of this particle while the direct production of IDL was increased (113).

### Insights From the Mechanism of Action of Statins and PCSK9 Inhibitors

It has been recognized for some time that statins have the capacity to reduce plasma triglyceride concentrations to a modest degree in individuals with normal triglyceride levels and more substantially in those with elevated concentrations. A “rule-of-thumb” has emerged that in hypertriglyceridemic subjects, the percentage reduction in plasma triglyceride equals that of LDL cholesterol (114). Metabolic studies investigating the mechanism by which statins lowered VLDL in hypertriglyceridemic subjects revealed that the drug stimulated the fractional clearance rates of all apoB-containing lipoproteins from VLDL_1_ through to LDL (115–118). These observations are consistent with the concept that receptors, especially the LDL receptor, play a critical role in catabolism of lipoproteins across the VLDL-LDL metabolic cascade as depicted in Figure 3. VLDL_1_ direct catabolism does not seem to involve the LDL receptor pathway (Figure 3) and yet it was accelerated in subjects on statin therapy. A possible explanation is that another lipoprotein receptor, regulated by cellular cholesterol in a manner similar to the LDL receptor is stimulated by statins in hypertriglyceridemic subjects (Figure 5). One possibility is LRP1 which is reported to be upregulated by statins (119).

Evidence from clinical trials indicates that reduction in plasma triglyceride and VLDL on PCSK9 inhibitors occurs but is less marked than that seen when a statin is given, even when the drugs are used as monotherapy (118, 120, 121). Kinetic studies conducted to date show small changes if any in VLDL apoB metabolism when PCSK9 inhibitors are added to background statin therapy or used alone (118, 120); the most notable perturbation is a modest increase in VLDL apoB FCR. Exploration of the action of PCSK9 inhibitors on chylomicron metabolism shows a lack of effect on the post prandial rise in triglyceride following a fat meal challenge, and on the increment in apoB48 levels (118, 121). However, these investigations were conducted in subjects with low or normal plasma triglyceride and it might be expected that the response would be more marked in subjects with raised plasma triglyceride (as for statins). In a recent study in patients with diabetes, it was observed that plasma triglyceride was reduced about 15% (122), and we saw a similar, small but significant drop in diabetic patients with borderline raised triglyceride treated with evolocumab that was accompanied by a decrease in apoCIII, a reduced post-prandial rise in triglyceride, a 29% decrease in remnant cholesterol, and a 17% lower increment in apoB48 following a standard fat meal (123). We found further that evolocumab appeared to have a significant impact on the fasting triglyceride concentration in VLDL_2_ (23% decrease, *P* < 0.001) but not in VLDL_1_. A tentative interpretation of this unusual finding—normally when triglyceride is lowered the effect on VLDL_1_ is greater than that on VLDL_2_–is that PCSK9 inhibitors do not impact on the activity of LRP1 (Figure 5) since this receptor lacks the binding motif for PCSK9 and so is not regulated by this protein (62), but VLDL_2_ removal by the LDL receptor is stimulated.

## Conclusions

The potential benefits for cardiovascular disease prevention of triglyceride lowering were equivocal in trials of both fibrates and omega-3 fatty acids, at least prior to REDUCE-IT (1–3, 12, 100, 108). This was attributed to either the possibility that the target—triglyceride rich lipoprotein—was not part of the causal pathway, or that the wrong patient phenotype had been selected, or that the agents did not have sufficient impact on correcting the underlying dyslipidaemia. With the positive results of the first large-scale outcome trial to focus on hypertriglyceridemic subjects (12), and the persistent *post-hoc* observation that individuals with a phenotype of raised triglyceride and low HDL cholesterol do appear to benefit in terms of relative risk reduction in the major fibrate trials (3), it may be that triglyceride-lowering to be effective needs to be deployed in people with the hypertriglyceridemia-driven disturbances in apoB metabolism described in this review. This is true also when triglyceride reduction is used as added treatment on top of statin therapy since the association with risk persists even though LDL cholesterol levels are well-managed (124, 125). It is clear that in the hypertriglyceridemic state abnormalities exist in a number of metabolic pathways and that elevation in triglyceride levels is accompanied by perturbations throughout the VLDL_1_-VLDL_2_-IDL-LDL delipidation cascade. Deciphering the quantitative relationship between drug (or diet)—induced decrease in plasma triglyceride and reduced risk of ASCVD, as has been elegantly done for the much simpler case of LDL cholesterol lowering, may prove challenging. If the critical change is in the circulating concentrations of VLDL remnants, chylomicron remnants, and specific LDL subfractions (Figure 2) then better analytical measures are needed to quantify these lipoprotein species. Finally, it is recognized increasingly that the size of the unmet clinical need is substantial in the “real-world” where surveys have revealed a high prevalence of persistent hypertriglyceridemia in well-treated patients with established ASCVD (126), and in patients with diabetes and metabolic syndrome (127, 128).

## Author Contributions

All authors listed have made a substantial, direct and intellectual contribution to the work, and approved it for publication.

## Conflict of Interest

CP claims grants and honoraria from MSD, Daiichi-Sankyo, Amgen. M-RT claims grants and honoraria from Amgen, Chiesi Pharma, Sanofi-Aventis, NovoNordisk, Mylan. The authors declare that the research was conducted in the absence of any commercial or financial relationships that could be construed as a potential conflict of interest.
